# Reverse and Conventional Chemical Ecology Approaches for the Development of Oviposition Attractants for *Culex* Mosquitoes

**DOI:** 10.1371/journal.pone.0003045

**Published:** 2008-08-22

**Authors:** Walter S. Leal, Rosângela M. R. Barbosa, Wei Xu, Yuko Ishida, Zainulabeuddin Syed, Nicolas Latte, Angela M. Chen, Tania I. Morgan, Anthony J. Cornel, André Furtado

**Affiliations:** 1 Honorary Maeda-Duffey Laboratory, Department of Entomology, University of California Davis, Davis, California, United States of America; 2 Departamento de Entomologia, Centro de Pesquisas Ageu Magalhaes-Fiocruz, Recife, Brazil; 3 Mosquito Control Research Laboratory, Department of Entomology, University of California Davis, Davis, California, United States of America; University of Arizona, United States of America

## Abstract

Synthetic mosquito oviposition attractants are sorely needed for surveillance and control programs for *Culex* species, which are major vectors of pathogens causing various human diseases, including filariasis, encephalitis, and West Nile encephalomyelitis. We employed novel and conventional chemical ecology approaches to identify potential attractants, which were demonstrated in field tests to be effective for monitoring populations of *Cx. p. quinquefasciatus* in human dwellings. Immunohistochemistry studies showed that an odorant-binding protein from this species, CquiOBP1, is expressed in trichoid sensilla on the antennae, including short, sharp-tipped trichoid sensilla type, which house an olfactory receptor neuron sensitive to a previously identified mosquito oviposition pheromone (MOP), 6-acetoxy-5-hexadecanolide. CquiOBP1 exists in monomeric and dimeric forms. Monomeric CquiOBP1 bound MOP in a pH-dependent manner, with a change in secondary structure apparently related to the loss of binding at low pH. The pheromone antipode showed higher affinity than the natural stereoisomer. By using both CquiOBP1 as a molecular target in binding assays and gas chromatography-electroantennographic detection (GC-EAD), we identified nonanal, trimethylamine (TMA), and skatole as test compounds. Extensive field evaluations in Recife, Brazil, a region with high populations of *Cx. p. quinquefasciatus*, showed that a combination of TMA (0.9 µg/l) and nonanal (0.15 ng/µl) is equivalent in attraction to the currently used infusion-based lure, and superior in that the offensive smell of infusions was eliminated in the newly developed synthetic mixture.

## Introduction

Mosquitoes in the genus *Culex* are major vectors of pathogens causing human diseases throughout the world, including *Wulchereria bancrofti* and arboviruses, such as, St. Louis encephalitis (SLE), Japanese encephalitis (JE), Venezuelan equine encephalitis (VEE), Western equine encephalitis (WEE), and West Nile Virus (WNV) [1]. Surveillance and control programs have reduced the threat from endemic SLE in California and now mitigate the impact of WNV invasion [2]. Monitoring mosquito populations and mosquito-borne virus activity are the cornerstones of surveillance programs. Although CDC-style CO_2_ traps can be very effective tools for monitoring mosquito populations, the majority of mosquitoes collected in these traps have not taken a bloodmeal [3]. In theory sampling with gravid traps should represent a more efficient surveillance tool because they collect proportionately more parous and gravid females that have taken bloodmeals and thus have had the opportunity to become horizontally infected [4], [5]. One of the major disadvantages of the gravid traps, however, is the use of cumbersome, infusion-based attractants whose offensive smell hinders population monitoring in human dwellings. This prompted us to undertake a multi-disciplinary approach to explore the development of user-friendly, chemically-based lures for gravid females in the *Culex pipiens* complex. First, we examined the expression pattern of an odorant-binding protein (OBP), CquiOBP1, which was first isolated from the Southern House mosquito, *Cx. p. quinquefasciatus* [6] and later determined to be homologous to a female antennae-specific OBP from *Cx. tarsalis* and identical to OBPs from *Cx.p. pipiens* and *Cx. p. molestus* (Ishida and Leal, EU723597 and EU723598). Having observed that CquiOBP1 binds to a previously identified mosquito oviposition pheromone (MOP) in a pH-dependent manner, we used CquiOBP1 as a molecular target to identify a candidate compound. Then, we identified electrophysiologically-active compounds from rabbit chow infusion and conducted extensive field tests to develop a simple and convenient synthetic attractant lure for trapping gravid females of the Southern house mosquito.

## Results and Discussion

### Expression of CquiOBP1

**Figure 1:**
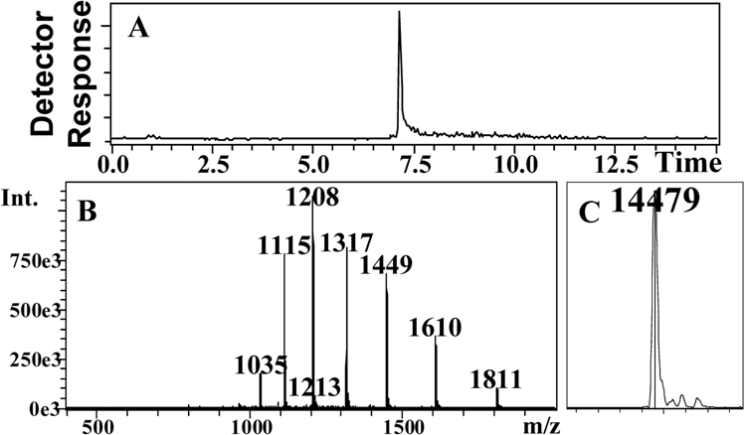
Mass spectral data for purified recombinant CquiOBP1. (A) HPLC separation, (B) mass spectrum of CquiOBP1 peak, and (C) deconvolution data indicating a molecular mass of 14,479 Da.

**Figure 2:**
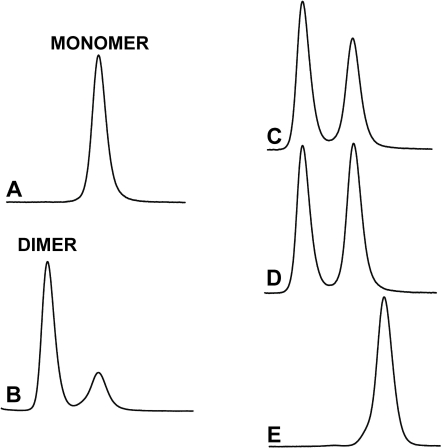
Gel filtration elution profiles of CquiOBP1. (A) Monomeric and (B) dimeric form of CquiOBP1. The dimer was isolated with a minor peak of the monomer. The dimer dissociates into monomer as indicated by the increase in the second peak (C) after 1 hour at room temperature and (D) overnight at 4°C. The dimeric form is also dissociated with organic solvent. (E) Sample D analyzed with acetonitrile in the mobile phase.

Recombinant CquiOBP1 was prepared by a perisplamic expression system, which is known to generate properly folded, functional OBPs [7]. Purification by a combination of ion-exchange chromatography and gel filtration generated samples of high purity, as indicated by LC-ESI/MS analysis (Fig. 1). The MS data also suggest that all 6 cysteine residues in CquiOBP1 are linked to form three disulfide bonds (observed, 14,479 Da; calculated, 14,486 Da or 14,480 with disulfide bridges). Formation of three disulfide bridges have also been shown in a homologous protein [8], AgamOBP1, an OBP cloned from the malaria mosquito, *Anopheles gambiae* sensu stricto [9]. During gel filtration separations, recombinant CquiOBP1 samples were isolated in monomeric and dimeric forms (Fig. 2), but the dimer slowly dissociated into the corresponding monomer. Because we did not observe dimerization of the isolated monomer and considering that rCquiOBP1 monomer migrated in native gel as the native protein (data not shown), we used only the monomeric form of the protein in subsequent studies.

### Localization of CquiOBP1 in olfactory sensilla of *Cx. p. quinquefasciatus*

CquiOBP1 was the first OBP isolated from any mosquito species [6]. Since then homologous proteins have been isolated and/or cloned from the malaria mosquito, *A. gambiae* s. s. [9], *Cx. tarsalis* [10], *Cx. pipiens* and *Cx. molestus* (Ishida and Leal, EU723597 and EU723598), and the yellow fever mosquito, *Aedes aegypti* [11], [12]. Although CquiOBP1 was demonstrated to be expressed specifically in female antennae of the Southern house mosquito, OBPs from mosquitoes are yet to be mapped in specific olfactory tissues.

**Figure 3:**
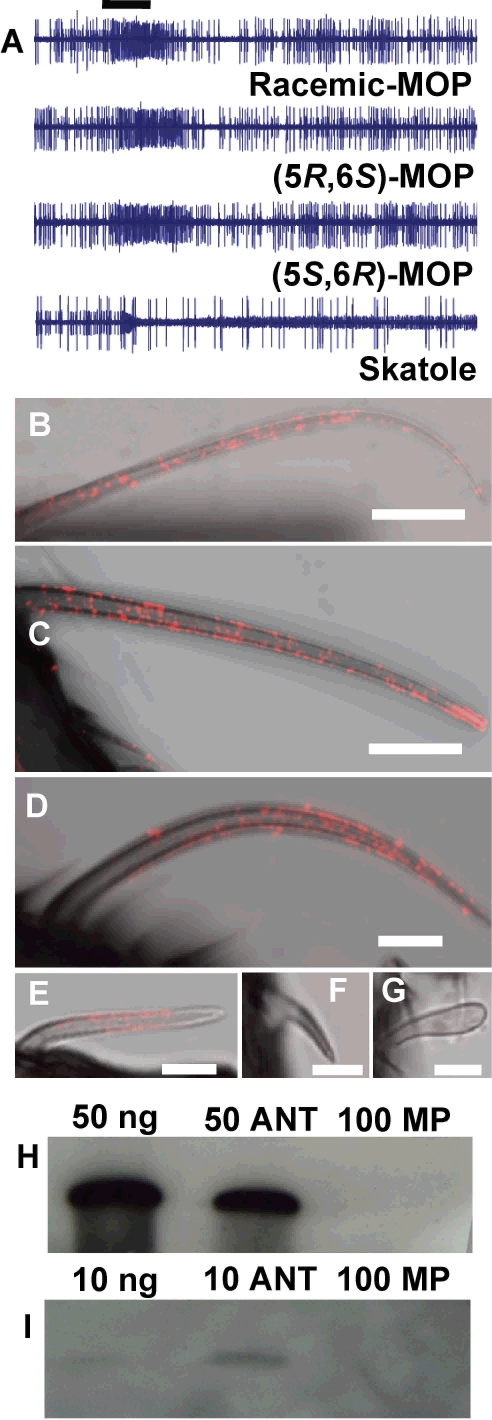
Oviposition Pheromone Reception. (A) Single sensillum recordings from short, sharp-tipped trichoid sensilla on the antennae of female *Cx. quinquefasciatus*. Response of a neuron to both the natural stereoisomer, (5*R*,6*S*)-MOP, and its antipode. The sensilla housed a second olfactory receptor neuron, characterized by a smaller spike amplitude, which was very sensitive to skatole. Bar denotes stimulus duration, 500 ms. Immunohistochemical localization of CquiOBP1 in the trichoid (B) long, sharp-tipped sensilla, (C) long, sharp-tipped sensilla with high density labeling at the excised tip, (D) MOP-detecting short, sharp-tipped sensilla, and (E) blunt-tipped sensilla on the antennae of female *Cx. quinquefasciatus*. CquiOBP1 was not detected in the (F) grooved peg sensilla on the antennae, and (G) the peg sensilla on maxillary palps. Scale bars, B, C: 10 µm, others, 5 µm. (H) Western blot analysis of protein extracted from olfactory tissues compared to recombinant CquiOBP1. (I) Same analysis as in H, but with 5× lower amounts of rCquiOBP1 and antennal extract. ANT, antenna-equivalent; MP, maxillary palp-equivalent. While signal was detected from 10 antenna-equivalent, no signal was observed from extracts of 100 maxillary palp-equivalent.

The olfactory sensilla in *Culex* mosquitoes are morphologically comparable to those in *A. aegypti* [13]. The antennae are endowed with three types of trichoid (single-walled multiporous) sensilla and one type of grooved (double-walled multiporous) peg sensilla, whereas the maxillary palps house only (single-walled multiporous) peg sensilla. The trichoid sensilla are further classified into sharp-tipped (A1), long (A1-I), or short (A1-II), and blunt-tipped (A2), whereas the grooved pegs are designated as A3. Single-sensillum recordings indicate that MOP is detected by an olfactory receptor neuron with large spike amplitude (Fig. 3A) in the short, sharp-tipped trichoid (A1-II) sensilla. Although these sensilla are morphologically indistinguishable, we identified two types of A1-II sensilla involved in MOP reception, a more frequently encountered type which responded with nearly the same sensitivity to both enantiomers of MOP (Fig. 3A) and a more rare type that responded only to the compound with the same stereochemistry as the natural pheromone, (5*R*,6*S*)-MOP [14]–[16], and was silent to its antipode (data not shown). Both types of sensilla housed also a small spike amplitude neuron, which was very sensitive to skatole, a compound previously identified as a mosquito oviposition attractant [17], [18]. Although we found olfactory receptor neurons sensitive to (5*S*,6*R*)-MOP, behavioral and field evaluations indicated that the non-natural stereoisomer is neither an attractant nor repellent [14]–[16], [19]–[22].

Immunohistochemistry (IHC) with affinity purified CquiOBP1-specific antibody provided data on the precise localization of CquiOBP1 in *Cx. p. quinquefasciatus* olfactory tissues. Labeling indicated that CquiOBP1 is expressed in most types of sensilla on the antennae, but not in the grooved pegs on the maxillary palps (Fig. 3B–3G). Fluorescence signal showed the presence of CquiOBP1 in the long, sharp-tipped sensilla (A1-I) (Fig. 3B), the short, sharp-tipped (A1-II) sensilla (Fig. 3D), and blunt-tipped (A2) sensilla (Fig. 3E). Sensilla fractured or cut during preparations showed high density labeling at the exposed site suggesting that these incisions allowed more penetration of antibody (Fig. 3C), whereas sensilla from control treatments showed no labeling (data not shown). Density of labeling in the sensillar lymph of grooved pegs (A3) sensilla (Fig. 3F) was below the detection limit. We concluded that CquiOBP1 is not expressed in the A3 sensilla, but a caveat is the possibility that the double-walled structures of these sensilla filter out fluorescence signal. No density labeling was observed in the single-walled multiporous peg sensilla (Fig. 3G) on the maxillary palps, but in this case we were able to unambiguously demonstrate that CquiOBP1 is not expressed in the maxillary palps. Western blot analyses (Fig. 3H and 3I) confirmed that, as opposed to antennae, the maxillary palps express no detectable amounts of CquiOBP1. Previously, we have shown that the grooved peg sensilla on the maxillary palps are more than CO_2_ detectors and house olfactory receptor neurons, which are highly sensitive to 1-octen-3-ol, and various plant-derived compounds [23]. Therefore, CquiOBP1 is unlikely to be involved in the transport of any of these odorants in the sensillar lymph of the peg sensilla on the maxillary palps. On the other hand, expression of CquiOBP1 in all types of antennal trichoid sensilla suggests that this olfactory protein may be involved in the detection of MOP and other semiochemicals.

### pH-Dependent binding of MOP to CquiOBP1

**Figure 4:**
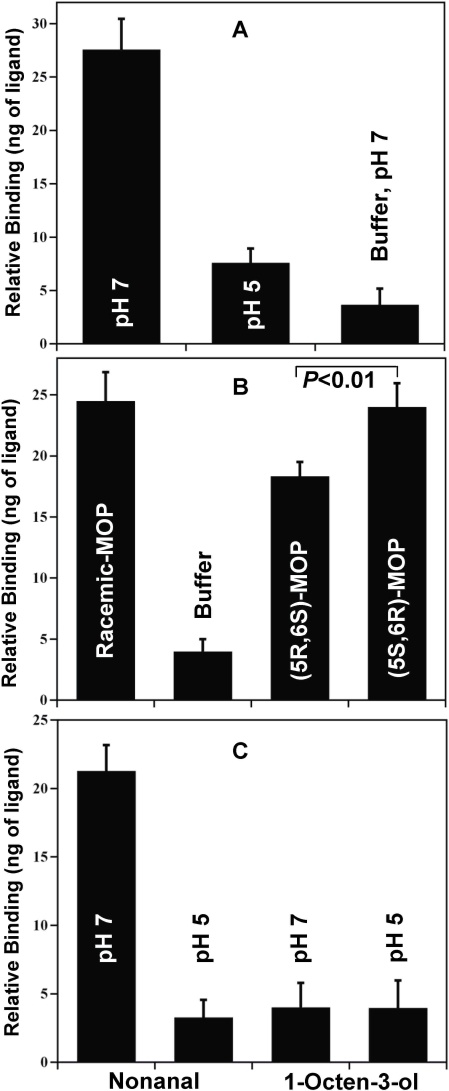
Binding of test ligands to antennae-specific CquiOBP1. (A) pH-dependent binding of racemic MOP to CquiOBP1. (B) Binding of enantiomers compared to racemic MOP. The non-natural stereoisomer, (5*S*,6*R*)-MOP showed significantly higher affinity for CquiOBP1 than the natural pheromone. (C) Nonanal bound to CquiOBP1 with high affinity at high but not low pH, whereas 1-octen-3-ol did not bind the protein at high or low pH.

**Figure 5:**
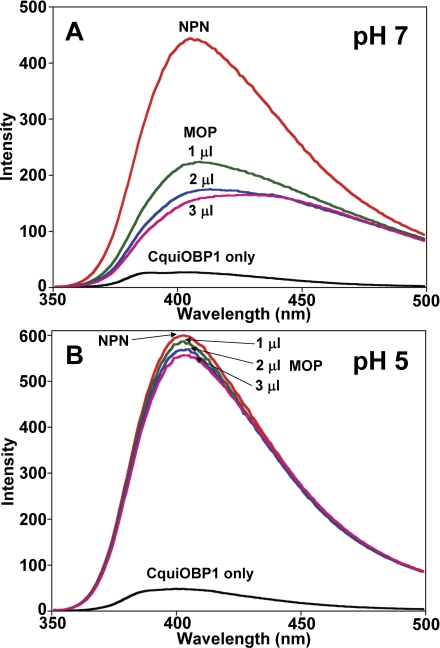
Competitive binding of MOP to CquiOBP1. Fluorescence emission spectra of CquiOBP1 alone (15 µg/ml; black), in the presence of NPN (2 µl, 3.2 mM; light red), and after titrating with increasing amounts of MOP (1–3 µl, 3.2 mM; green, blue, and dark red lines). (A) Replacement of fluorescent reporter by MOP is indicated by decrease of emission, and suggests competitive binding. (B) Excitation of the fluorescent reporter was not changed with addition of MOP thus indicating no MOP binding at low pH.

**Figure 6:**
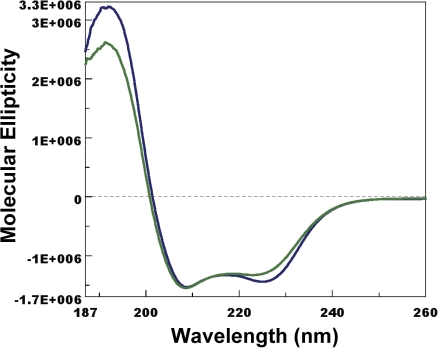
Far-UV circular dichroism spectra of CquiOBP1 at pH 6.5 (blue) and pH 5 (green). The helical-rich protein underwent unwinding of α-helix at low pH as indicated by the change in the intensity of the second minima.

Given the high level of CquiOBP1 expression in antennae, we reasoned that it might be involved in the detection of MOP and other oviposition attractants. Indeed, MOP binds to CquiOBP1 with apparently high affinity at pH 7 (Fig. 4A). Since moth pheromone-binding proteins undergo pH-dependent conformational changes [7], [24] that leads to decreased binding affinity at low pH [25], [26], we examined the effect of pH on the ability of CquiOBP1 to bind MOP. At low pH, the amount of ligand recovered by incubation with protein was not significantly different from that detected in buffer only (Fig. 4A) (Wilcoxon-Mann-Whitney unpaired rank sum test, *P*>0.05) thus suggesting that at low pH MOP does not bind to CquiOBP1, or binds with significantly reduced affinity. To examine further the pH-dependent binding of CquiOBP1 to MOP, we tested binding at high and low pH by fluorescence using NPN as a fluorescent reporter [27]. MOP replaced NPN at pH 7 (Fig. 5A), but not at pH 5 (Fig. 5B) thus confirming that binding affinity is lost or is very weak at low pH. The molecular basis for loss of binding affinity of pheromones to pheromone-binding proteins at low pH has been elucidated in moths [7], [24], [28]–[30]. Protonation of acidic residues in the C-terminus of the silkworm pheromone-binding protein at low pH triggers the formation of an additional α-helix [29], [31], which occupies the binding cavity [28]. By contrast, the structure of AgamOBP1 [8], a protein homologous to CquiOBP1, lacks a corresponding C-terminal helix that is found in the moth protein. Therefore, CquiOBP1 may have a different mechanism for pH-dependent odorant binding. Circular dichroism data suggest that the helical content of CquiOBP1 is reduced at low pH (Fig. 6) thus implying possible unwinding of helical structure(s) at low pH.

**Figure 7:**
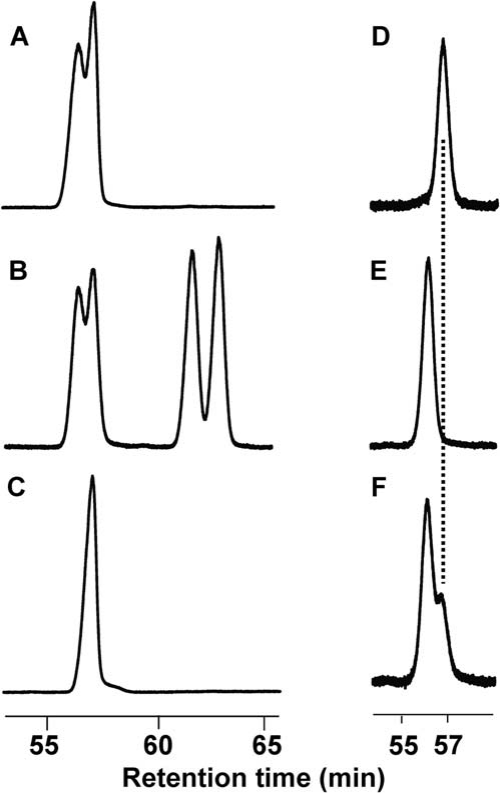
Resolution of MOP stereoisomers on a chiral column. (A) Partially resolved enantiomers of MOP. (B) Random mixture of stereoisomers showing two clusters of well separated stereoisomers: erythro- and threo-MOP. (C) The pheromone isolated from egg rafts of *Cx. quinquefasciatus* showed the same retention time as the second peak in the erythro-MOP cluster, and is thus confirmed to be (5*R*,6*S*)-MOP. (D) The natural stereochemistry and (E) the configuration of the antipode were retained upon binding. (F) Competitive binding with the two enantiomers showed that CquiOBP1 has a higher affinity for the non-natural stereoisomer (first peak) than for natural stereoisomer, (5*R*,6*S*)-MOP (the second peak).

Next, we examined if CquiOBP1 could discriminate MOP enantiomers. The stereochemistry of the natural product isolated from eggs of *Culex pipiens fatigans* ( = *Cx. p. quinquefasciatus*) has been determined to be (5*R*,6*S*)-6-acetoxy-5-hexadecanolide, [(5*R*,6*S*)-MOP] [16]. We isolated the oviposition pheromone from the Southern house mosquito, analyzed the extract by GC with a chiral column and confirmed that *Cx. p. quinquefasciatus* produces (5*R*,6*S*)-MOP (Fig. 7C) thus ruling out possible geographical or other variations. Binding assays showed that both stereoisomers of MOP bound to CquiOBP1 at pH 7 (Fig. 4B), but not at pH 5 (data not shown). Intriguingly, the antipode of the natural compound, (5*S*,6*R*)-MOP showed significantly higher binding affinity than the natural pheromone (Wilcoxon-Mann-Whitney unpaired rank sum test, *N* = 15, P<0.01) (Fig. 4B). Because this assay [26] is sensitive to the amount of ligand incubated, it is worth mentioning that the amounts of the enantiomers were adjusted by GC to measure binding to analytically equal amounts of the two enantiomers. This chiral mismatch of MOP and CquiOBP1 suggests that odorant receptor(s) may play a more significant role than CquiOBP1 in the chiral discrimination observed by olfactory receptor neurons in one type of A1-II sensilla (data not shown). An inability to discriminate enantiomers has also been observed in the pheromone-binding protein from the Japanese beetle [32].

We determined the stereochemistry of ligands recovered in these binding assays by GC. It was not surprising that both the natural stereoisomer and its antipode retained their absolute configuration. When synthetic (5*R*,6*S*)-MOP was incubated with CquiOBP1 the pheromone with the same configuration as the natural product was recovered from the bound protein (Fig. 7D). Likewise, the antipode retained its configuration and was recovered as (5*S*,6*R*)-MOP (Fig. 7E). Interestingly, a racemic mixture of MOP extracted from bound-CquiOBP1 had a much higher proportion of the non-natural stereoisomer (Fig. 7F). In these enantiocompetitive assays, despite the protein being incubated with a racemic mixture slightly richer in the natural stereoisomer (Fig. 7A) more (5*S*,6*R*)-MOP was still recovered from the bound protein. Inversion of configuration can be ruled out on the basis of experiments with one enantiomer at a time. Taken together, these data suggest that CquiOBP1 has a higher affinity for (5*S*,6*R*)-MOP than the enantiomer with the same absolute configuration as the oviposition pheromone.

### Prospecting for oviposition attractants

Since the mosquito oviposition pheromone is detected only by antennae and CquiOBP1 is likely involved MOP reception (see above), we used this molecular target in a “reverse chemical ecology” approach to identify candidate compound(s) for subsequent field tests. We measured binding of CquiOBP1 to a few compounds known/inferred to be mosquito attractants. While CquiOBP1 bound to nonanal with apparently high affinity, we detected no binding to 1-octen-3-ol (Fig. 4C), 1-octyn-3-ol, (*R*)-4-isopropenyl-1-methylcyclohexene (d-limonene), and oxidized d-limonene (data not shown). Attempts to determine binding of CquiOBP1 to skatole and cresols were unsuccessful, because high levels of non specific binding generated high backgrounds after incubation even with buffer alone. The fact that 1-octen-3-ol is detected with high sensitivity by olfactory receptor neurons in the maxillary palps [23] is consistent with both the results of our binding assays and the lack of CquiOBP1 expression in peg sensilla on the maxillary palps (Fig. 3G). This olfactory protein-based approach prompted us to test nonanal in the field and not to explore 1-octen-3-ol as an oviposition attractant. The latter compound has been demonstrated to attract other *Culex* species, but not *Cx. p. quinquefasciatus* [33], and furthermore it is unlikely to be an oviposition attractant. On the other hand, it has been demonstrated in laboratory bioassays that *Cx. p. quinquefasciatus* laid more eggs in water treated with candidate compound, nonanal, than in controls [17].

Field tests showed that gravid female traps baited with nonanal at all concentrations tested caught significantly more *Cx. p. quinquefasciatus* females than control traps, but the efficacy of the traps was dose-dependent. Catches in traps loaded with 0.15 ng/µl of nonanal (mean±SEM, 19.1±2.2 female/trap/night) were significantly higher (*N* = 12, Tukey HSD, *P*<0.01) than captures in control water traps (2.5±0.6 female/trap/night), and did not differ significantly from catches in infusion-baited traps (25.2±1.2 female/trap/night). Captures decreased at higher and lower concentrations: 15 ng/µl (6.6±1.4 female/trap/night), 1.5 ng/µl (7.5±1.1 female/trap/night), and 0.015 ng/µl (7.7±1.9 female/trap/night).

### GC-EAD-based identification of attractants from rabbit chow fermentation

**Figure 8:**
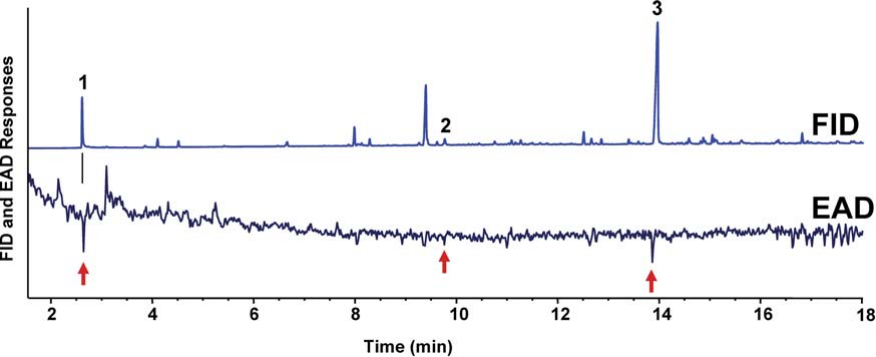
GC-EAD analysis of rabbit chow fermentation products. The three EAD-active peaks (red arrows) were identified as (1) trimethylamine, (2) nonanal, and (3) skatole.

We further prospected for test compounds by a conventional chemical ecology approach, i.e., gas chromatography coupled with an electroantennographic detector (GC-EAD). In a project concurrent with this work, we have evaluated infusion-based gravid traps in California, and observed that proportionally more WNV-infected *Culex* mosquitoes were collected in gravid traps than in CO_2_-baited traps, although total capture of mosquitoes in the latter was significantly higher than catches in traps baited with Bermuda grass or rabbit chow infusions [34]. Of particular note, rabbit chow-baited traps collected the largest numbers of urban *Cx. p. quinquefasciatus* mosquitoes in Los Angeles, even outperforming CO_2_ traps. We, therefore, aimed at identifying electrophysiologically-active compounds from rabbit chow fermentations using GC-EAD, a technique previously utilized to identify attractants from Bermuda grass infusions [17]. With solid-phase micro extraction (SPME), three EAD-active peaks (Fig. 8) were detected, including a compound with short retention time that probably would have been masked by the solvent peak in a conventional solvent extraction. Compounds 1, 2, and 3 were first identified by GC-MS and then confirmed with authentic standards to be trimethylamine (TMA), nonanal, and 3-methylindole (skatole), respectively.

### In-depth field evaluation of *Cx. p. quinquefasciatus* attractants

Long-term field experiments to evaluate TMA and skatole individually as well as in binary and tertiary mixtures, including combinations with nonanal were conducted in Recife, Brazil. As opposed to field experiments with agricultural pests, evaluation of mosquito oviposition attractants is a time-consuming task due to heterogeneity and daily fluctuations of field populations, and, consequently, the need for high number of replicates to generate statistically reliable data. With the molecular and GC-EAD approaches we were able to concentrate our efforts on three potential attractants. First, we tested if individual compounds were attractants and then aimed at determining the optimal doses. The highest trap catches with TMA-baited gravid traps were obtained with 0.9 µg of TMA per liter (7.5±1.3 females/trap/night), which was significantly higher than captures in control (water) traps (1.3±0.5 females/trap/night; *N* = 5, Tukey HSD, *P*<0.01). Captures decreased dramatically at lower concentrations of 90 ng/L (4.1±1.1 females/trap/night), 9 ng/L (3.1±1.2 females/trap/night), and 0.9 ng/L (2.3±0.9 females/trap/night). Catches in traps baited with a higher than optimal concentration, 9 µg/L (1.7±0.5 females/trap/night), did not differ significantly from those in control traps (*P*>0.05). Previous laboratory and field assays showed that *Aedes albopictus* did not exhibit attraction, greater oviposition, or an electrophysiological response to TMA [35]. We also observed concentration-dependent performance of skatole-baited traps, in agreement with previously field studies [18]. Captures in traps baited with skatole in decadic solutions (20 pg to 0.2 µg of skatole per liter) were all significantly greater than catches in control traps (2.5±0.8 female/trap/night), with the greatest captures being obtained with 2×10^−3^ µg/L (9.8±2.1 females/trap/night) and 20×10^−3^ µg/L (7.4±1.2 females/trap/night). Because these two dosages were not significantly different (Tukey HSD, *P*>0.05), the higher concentration (20×10^−3^ µg/L) was adopted to compensate for evaporation in week-long experiments. The dose-dependence observed in our field tests is consistent with previous studies [18], but the optimal doses differ, probably because of differences in trap designs and, more importantly, differences in end-point measurements. While we evaluated performance on the basis of female capture, Mboera and collaborators focused on egg-laying by counting the number of egg rafts.

**Figure 9:**
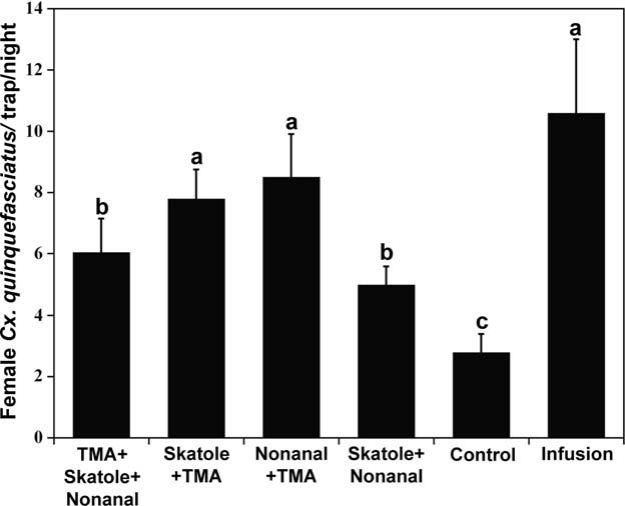
Field data comparing captures of female *Cx. quinquefasciatus* in synthetic mixtures- and infusion-baited traps. Catches in traps baited with nonanal and TMA, skatole and TMA, and infusion were not significantly different (Tukey HSD, *P*>0.05), but the nonanal plus TMA lure is odorless and thus suitable for surveillance and use in monitoring population in human dwellings.

Tests with binary mixtures showed that a combination of skatole and TMA (5.3±1.1 female/trap/night; *N* = 8) did not improve captures compared to traps baited with skatole (4.3±0.9 female/trap/night) or TMA alone (6.5±1.3 female/trap/night). By contrast, captures in traps baited with a combination of nonanal and skatole (13.9±1.9 female/trap/night; *N* = 17) were higher than those in traps with individual compounds (nonanal, 5.1±0.9; skatole, 10.1±2.7 female/trap/night). A synergistic effect was observed with a combination of nonanal and TMA (10.1±2.5 female/trap/night; *N* = 10) compared to TMA alone (2.7±0.6 female/trap/night) or nonanal alone (3.7±1.2 female/trap/night). Due to the difficulty of simultaneously testing a large number of lures (three compounds at 3–5 dosages), these tests with binary versus individual compounds were conducted at different times of the year with different population levels, but later the optimal binary mixtures were compared at the same time with a tertiary mixture and positive-control infusion. In these competitive field tests, captures in traps baited with binary or tertiary synthetic mixtures were significantly higher than those in negative control traps (Fig. 9). Catches in traps baited either with nonanal plus TMA or skatole plus TMA were not significantly different (Tukey HSD, *N* = 19; *P*>0.05) from captures in traps loaded with infusion (positive control). In the doses tested, the combination of the three compounds did not perform better than the binary mixtures. We adopted the mixture of nonanal and TMA for subsequent studies, because at the optimal concentrations this lure is odorless, performs comparably to infusion, and is suitable for use in human dwellings.

**Figure 10:**
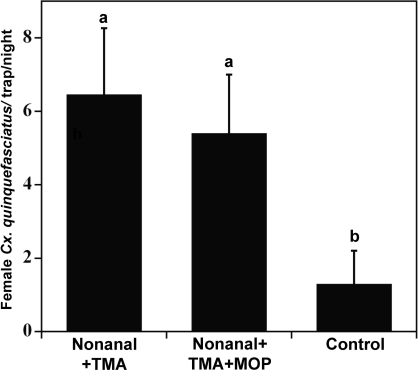
Catches in traps baited with a synthetic attractant alone or in combination with MOP. Captures in traps loaded with pheromone were not significantly different (Tukey HSD, *N* = 15, *P*>0.05) from those in trap baited only with nonanal plus TMA.

We then explored the possibility of synergism between this attractant mixture and MOP. Preliminary experiments with a previously tested dose (20 mg per trap) showed no significant difference between catches in traps baited with nonanal plus TMA compared to those with this binary mixture plus MOP. Follow-up experiments at lower doses demonstrated the same trend (Fig. 10). The apparent discrepancy between our data and those previously reported [18], [19], [36] might be related to differences in behavioral measurements. We evaluated MOP for attraction (i.e., numbers captured) of gravid females whereas previous reports measured the effect of MOP and other attractants on egg laying. While the former is more important for surveillance, the latter seems to be a more tangible measurement for controlling mosquito populations. The differences in these results suggest that MOP might be an arrestant rather than an attractant thus leading to increased egg laying but not necessarily gravid female capture.

In summary, this work is the first report of translational research combining molecular basis of olfaction and chemical ecology to generate deliverable material for medical entomology. We have employed an odorant-binding protein as a molecular target in binding assays in combination with a conventional chemical ecology approach to identify three compounds, namely, TMA, nonanal, and skatole, which were tested in extensive field studies. With this novel approach we developed a synthetic oviposition attractant mixture of nonanal and TMA, which is odorless and comparable in attraction to cumbersome infusions currently employed as standard lures. The newly developed user-friendly, synthetic mixture of readily available compounds holds considerable promise for future surveillance and management programs for *Cx. p. quinquefasciatus* and possibly other closely related *Culex* species with similar breeding requirements, which are major vectors of pathogens causing human diseases throughout the world.

## Materials and Methods

### Protein expression, purification, and antibody production

One microgram of pET-22b(+) vector (EMD Chemicals, Gibbstown, NJ) was digested with 6 U of *Msc* I (New England Biolabs, Ipswich, MA) at 37°C for 3 h. After purification of DNA by QIAquick PCR Purification Kit (Qiagen, Valencia, CA) the vector was digested with 7 U of *Bam* HI (New England Biolabs) at 37°C for 3 h and subsequently gel-purified by QIAquck Gel Extraction Kit (Qiagen). The following primers were used for amplification of insert DNA: 5CquiOBP-1-KpnI, 5′-GGGGTAC/ CCGACGTTACACCgCGTCGtGA-3′ and 3CquiOBP-1-BamHI, 5′-CGCG/ GATTCCTTAAACCAGGAAATAATGCT-3′. Slashes indicate cutting sites for *Kpn* I and *Bam* HI restriction enzymes, and lower cases in 5CquiOBP1-KpnI primer indicate bases replaced to overcome codon bias of *E. coli* and thus enhance protein expression. After amplification, and confirmation by sequencing, 16 µg of DNA was initially digested with 40 U of *Kpn* I (New England Biolabs) at 37°C for 3 h, purified by QIAquick PCR Purification Kit, blunted by T4 DNA polymerase (New England Biolabs) with dNTP, and purified again by QIAquick PCR Purification Kit. Then, the DNA was digested with 20 U of *Bam* HI at 37°C for 3 h and, gel- purified by QIAquick Gel Extraction Kit, and ligated into prepared pET vector by T4 DNA ligase (New England Biolabs). CquiOBP1 was expressed in LB medium with transformed BL21 (DE3) cells, according to a protocol for perisplasmic expression of OBPs [7]. Proteins in the periplasmic fraction were extracted with 10 mM Tris·HCl, pH 8 by three cycles of freeze-and-thaw [37] and centrifuging at 16,000 ×*g* to remove debris. The supernatant was loaded on a Hiprep™ DEAE 16/10 column (GE Healthcare Bio-Sciences, Piscataway, NJ). All separations by ion-exchange chromatography were done with a linear gradient of 0–500 mM NaCl in 10 mM Tris·HCl, pH 8. Fractions containing the target protein were further purified on a 20-ml Q-Sepharose Hiprep™ 16/10 column (GE Healthcare) and, subsequently, on a Mono-Q HR 10/10 column (GE Healthcare). OBP fractions were concentrated by using Centriprep-10 (Millipore, Billerica, MA) and loaded on a Superdex-75 26/60 gel-filtration column (GE Healthcare) pre-equilibrated with 150 mM NaCl and 20 mM Tris.HCl, pH 8. Highly purified protein fractions were concentrated by Centricon-10, desalted on four 5-ml HiTrap desalting columns (GE Healthcare) in tandem with water as mobile phase, and analyzed by LC-ESI/MS (see below), lyophilized, and stored at −80°C until use. The concentrations of the recombinant proteins were measured by UV radiation at 280 nm in 20 mM sodium phosphate, pH 6.5 and 6 M guanidine HCl by using the theoretical extinction coefficients calculated with EXPASY software (http://us.expasy.org/tools/protparam.html). An aliquot of highly purified CquiOBP1 was provided to Invitrogen (Camarillo, CA) for preparation of affinity purified CquiOBP1-specific rabbit antibody.

### Immunohistochemistry (IHC)

Immunofluorescence was performed with modification of previously published protocols [38], [39]. Heads of 5- to 7-day-old female *Cx. quinquefasciatus* were dissected from adult mosquitoes anesthetized on ice and fixed overnight with 4% paraformaldehyde in 1×PBS at 4°C. To dehydrate, the preparations were first rinsed in 1×PBS and then incubated overnight in 24% sucrose in 1×PBS at 4°C. After rinsing in 1×PBS, heads were embedded in Tissue Tec® optimal cutting temperature medium (Sakura Finetek, Torrance, CA) and frozen at −22°C on the object holder. Sections (14 µm) were prepared at −24°C (Leica CM1850 Cryostat, Bannockburn, IL) and after thawing they were mounted on Superfrost Plus slides (Fisher, Pittsburgh, PA) and air dried for at least 30 min. Preparations were incubated at 4°C initially in 4% paraformaldehyde in 1×PBS for 30 min, then in 1×PBS for 10 min, and finally in PBST for 30 min. After that, sections were washed twice for 5 min in 1×PBS and then the slides were incubated in PBST with 1% blocking reagent (Roche) for 30 min. Immunohistochemistry was performed using affinity purified CquiOBP1-specific rabbit antibody diluted 1∶1,000 in blocking solution. After washing 5 times with PBST, the sections were incubated with secondary antibody, Cy™3 linked goat anti-rabbit IgG (GE Healthcare, Piscataway, NJ) 1∶500 in blocking solution, for 1 h at room temperature in a humid box. Subsequently, slides were washed three times for 5 min each with PBS and sections were embedded in Vectashield mounting medium (Vector Laboratories, Burlingame, CA), covered and sealed with nail polish around the cover glass (Corning Labware and Equipment, Corning, NY). Confocal images were captured with a FV1000 Olympus Confocal Microscope system (Olympus America, Center Valley, PA).

### Western Blot

*Cx. p. quinquefasciatus* used in this study were from a laboratory colony originating from adult mosquitoes collected in Merced, CA in the 1950s and maintained under lab conditions at the Kearney Agricultural Center, University of California, as previously described [23]. Three to five-day-old adult mosquitoes were anesthetized on ice. Antennae and maxillary palps were dissected and homogenized in ice-cold glass homogenizers with 10 mM Tris-HCl, pH 8. Homogenized samples were centrifuged twice at 14,000 ×*g* for 10 min at 4°C. After concentration, the supernatants were analyzed by native and SDS polyacrylamide gel electrophoresis (15% PAGE), and proteins were electroblotted onto polyvinyl difluoride (PVDF) membranes (Bio-Rad Laboratories, Hercules, CA). After treatment with 1% blocking reagent (Roche, Indianapolis, IN) in 1×PBS (140 mM NaCl, 2.7 mM KCl, 1.8 mM KH_2_PO_4_, 10 mM Na_2_HPO_4_, pH 7.4) for 1 h at room temperature, the membrane was incubated for 1 h with affinity purified CquiOBP1-specific rabbit antibody diluted 1∶2,000 with 1% blocking reagent. After washing four times with PBST (1×PBS containing 0.1% Triton X-100), the membrane was incubated for 1 h with anti-rabbit IgG, horseradish peroxidase (HRP) conjugate (dilution 1∶5000) (Millipore, Temecula, CA). Immunoreacting bands were detected by treatment with SuperSignal West Femto Maximum Sensitivity Substrate (Pierce, Rockford, IL).

### Single sensillum recording

Recording from *Cx. p. quinquefasciatus* female antennae were performed as previously described for maxillary palps [23].

### Binding assays

Binding of CquiOBP1 to MOP and other test compounds was measured by incubating protein sample and test ligand, separating unbound and bound protein, extracting the bound ligand with hexane and quantifying by gas chromatography, according to a previously reported protocol [26]. pH-Dependent binding of CquiOBP1 to MOP was confirmed by an independent competitive binding assays using NPN as a fluorescent reporter [27].

### Chemicals

A random sample of the four isomers of MOP was prepared according to a previously reported method [20] and used only to determine the retention times of the four isomers after separation on a chiral column. Samples of racemic MOP, (5*R*,6*S*)-6-acetoxy-5-hexadecanolide, and (5*S*,6*R*)-6-acetoxy-5-hexadecanolide were gifts from Bedoukian Research Incorporated (BRI). The ^1^H and ^13^C NMR spectra of the racemic pheromone were consistent with published characterization data [40]. ^1^H NMR (400 MHz) 0.83 (t, 3H), 1.16–1.36 (br m, 16H), 1.53–1.66 (m, 3H), 1.72–1.98 (m, 3H), 2.04 (s, 3H), 2.35–2.46 (m, 1H), 2.51–2.60 (m, 1H), 4.28–4.34 (m, 1H), 4.91–4.97 (m, 1H); ^13^C (100 MHz) 14.12, 18.26, 21.03, 22.68, 23.48, 25.26, 29.31, 29.40, 29.45, 29.52, 29.54, 29.56, 29.63, 31.89, 74.26, 80.51, 170.45, 170.87. The enantiomeric purity of the two stereoisomers was determined by gas chromatography equipped with a chiral column, Chiraldex GTA (25 m×0. 25 mm; 0.125 µm; Astec, Whippany, NJ), which was operated at constant temperature, 175°C. The enantiomeric excess of (5*R*,6*S*)- and (5*S*,6*R*)-MOP were estimated to be 99.5 and 99%, respectively. For binding assays, samples of the stereoisomers of MOP were first prepared by weighting the amounts of the compounds and then their concentrations were adjusted to have the same amounts of the two stereoisomers by gas chromatography. Eicosyl acetate was a gift from Fuji Flavor Co., Tokyo, Japan and racemic 1-octen-3-ol was a gift from BRI. Nonanal and TMA were purchased from Fluka (Buchs, Switzerland), NPN and skatole were from Aldrich Chemical Co. (St. Louis, MI).

### Chemical analysis

GC-EAD was done with a gas chromatograph (HP 5890, Agilent Technologies, Palo Alto, CA) equipped with transfer line and temperature control units (Syntech, Kirchzarten, Germany). The effluent from the capillary column was split into EAD and flame ionization detector (FID) in a 3∶1 ratio. Antennae from blood-fed female mosquitoes were placed in EAG probes of an AM-01 amplifier (Syntech) and held in place with Spectra 360 electrode gel (Parker Laboratories, Orange, NJ). The analog signal was fed into an A/D 35900E interface (Agilent Technologies) and acquired simultaneously with FID signal on an Agilent Chemstation. GC-MS was obtained on a 5973 Network Mass Selective Detector (Agilent Technologies). Both GC-EAD and GC-MS were equipped with the same type of capillary column (HP-5MS, 30 m×0.25 mm; 0.25 µm; Agilent Technologies). The temperature program started at 50°C for 1 min, increased at a rate of 10°C/min to 250°C, and held at this final temperature for 10 min. Both GCs were operated under splitless mode with the injection port at 230°C and purge time 2 min.

### Other analytical procedures

Fluorescence measurements were done on a spectrofluorophotometer (RF-5301, Shimadzu, Kyoto, Japan) at 25±1°C. Samples in 2-ml cell equipped with magnetic stir bar were excited at 337 nm, and the emission spectra were recorded from 350 to 500 nm, with emission and excitation slit widths of 1.5 and 10 nm, respectively. The spectra were obtained with the protein sample (CquiOBP1, 10 µg/ml in either 20 mM ammonium acetate, pH 7 or in 20 mM sodium acetate, pH 5) and after adding NPN (3.2 mM, 2 µl; final concentration, 3.2 µM) and MOP (3.2 mM, 1–3 µl; final concentrations, 1.6–4.8 µM). CD spectra were recorded with a Jasco J-810 spectropolarimeter (Easton, MD) with CquiOBP1 (25 µg/ml) either in 20 mM ammonium acetate, pH 6.5 or in 20 mM sodium acetate, pH 5. LC-ESI/MS was performed with a LCMS-2010 (Shimadzu, MD). High pressure liquid chromatography (HPLC) separations were carried out on a ZorbaxCB C8 column (150×2.1 mm; 5 µm; Agilent Technologies, Santa Clara, CA) with a gradient of water and acetonitrile plus 2% acetic acid as a modifier. The detector was operated with the nebulizer gas flow at 1.0 liters/min and the curved desolvation line and heat block at 250°C.

### Infusions

Rabbit chow infusions were prepared as previously reported [34]. For volatile collections, aliquots (20 ml) of fresh batches were transferred to 100-ml beakers. Volatile compounds were trapped from the headspace of each beaker with 3–4 SPME syringes introduced through a cover of parafilm (American National Can, Neenah, WI), with one of the syringes being used for GC-MS analysis. We employed SPME blue fibers (StableFlex™, 65 µm, polydimethylsiloxane/divinylbenzene partially crosslinked; Supleco, St. Louis, MI). Grass infusions for field tests were prepared by adding 30 g of fresh Indian goosegrass, *Eleusine indica* (Cyperales, Poaceae), to 2 l of water and incubating at 27±2°C for 7 days.

### Field tests

Preliminary field tests in Davis and Sacramento, CA were discontinued after aerial sprays in the area in the summer of 2005 to mitigate the levels of West Nile virus-infected mosquitoes. Follow-up field tests (January, 2006 to July, 2008) were conducted in Recife, Brazil, a city endemic for lymphatic filariasis, with abundant populations of *Cx. quinquefasciatus* that breed throughout the year [41]. Tests were conducted in the backyards of 6 residences with gravid mosquito traps (Bioquip, Rancho Dominguez, CA). To minimize inconsistencies observed in preliminary experiments due to variations in battery power and fan speed, traps were modified to run on AC power. Traps filled with 5 l of tap water were placed on the ground with an intertrap distance of at least 3 m. Traps were inspected and rotated every morning for a week, collecting chambers were replaced, and the trapped mosquitoes were counted after the collecting chambers were placed in a freezer for 10 min. All samples were identified morphologically and then confirmed as *Cx. p. quinquefasciatus* by PCR [42]. Test compounds were diluted by transferring each sample with a separate, disposable glass capillary to water in each trap's tub. Throughout the paper, concentrations refer to the final concentrations of the attractants after dilution in 5 l of water. When MOP was tested in combination with other attractants, the former was released from microscope cover slides (ca. 2×2 cm, Corning Labware and Equipment), which were allowed to float on each trap's tub water. Preliminary data were obtained with 20 mg of pheromone, a dosage previously tested in the field [18]. Each glass cover was prepared by transferring small aliquots of a hexane solution of either racemic- or (5*R*,6*S*)-MOP and letting the solvent evaporate until the desired amount (100 µl, 200 mg/ml) was loaded on the glass. Additional experiments were conducted with a lower dosage (2 mg; 20 µl, 100 mg/ml or racemic-MOP). Data were transformed to log (x+1) and analyzed by ANOVA and Tukey HSD (honestly significant differences).
